# Ubiquitin-Based Probes Prepared by Total Synthesis To Profile the Activity of Deubiquitinating Enzymes

**DOI:** 10.1002/cbic.201200497

**Published:** 2012-09-25

**Authors:** Annemieke de Jong, Remco Merkx, Ilana Berlin, Boris Rodenko, Ruud H M Wijdeven, Dris El Atmioui, Zeliha Yalçin, Craig N Robson, Jacques J Neefjes, Huib Ovaa

**Affiliations:** a Division of Cell Biology, The Netherlands Cancer InstitutePlesmanlaan 121, 1066 CX Amsterdam (The Netherlands)http://research.nki.nl/Ovaalab/; b Northern Institute for Cancer ResearchNewcastle University, Framlington Place, Newcastle upon Tyne, NE2 4HH (UK)

**Keywords:** activity-based protein profiling, deubiquitinating enzymes, fluorescent probes, solid-phase synthesis, ubiquitin

## Abstract

Epitope-tagged active-site-directed probes are widely used to visualize the activity of deubiquitinases (DUBs) in cell extracts, to investigate the specificity and potency of small-molecule DUB inhibitors, and to isolate and identify DUBs by mass spectrometry. With DUBs arising as novel potential drug targets, probes are required that can be produced in sufficient amounts and to meet the specific needs of a given experiment. The established method for the generation of DUB probes makes use of labor-intensive intein-based methods that have inherent limitations concerning the incorporation of unnatural amino acids and the amount of material that can be obtained. Here, we describe the total chemical synthesis of active-site-directed probes and their application to activity-based profiling and identification of functional DUBs. This synthetic methodology allowed the easy incorporation of desired tags for specific applications, for example, fluorescent reporters, handles for immunoprecipitation or affinity pull-down, and cleavable linkers. Additionally, the synthetic method can be scaled up to provide significant amounts of probe. Fluorescent ubiquitin probes allowed faster, in-gel detection of active DUBs, as compared to (immuno)blotting procedures. A biotinylated probe holding a photocleavable linker enabled the affinity pull-down and subsequent mild, photorelease of DUBs. Also, DUB activity levels were monitored in response to overexpression or knockdown, and to inhibition by small molecules. Furthermore, fluorescent probes revealed differential DUB activity profiles in a panel of lung and prostate cancer cells.

## Introduction

Ubiquitin (Ub) is a 76 amino acid protein regulator of a wide variety of cellular processes, including proteolysis by the proteasome, regulation of cell division, transcription regulation, and DNA repair. Ub modifies protein substrates by forming an isopeptide bond between the C-terminal carboxylate of Ub and the ε-amino moiety of a lysine side chain or the N terminus of the target protein or Ub itself.^1^ The conjugation of Ub is brought about by the consecutive action of Ub ligases from three classes, while Ub conjugates can be disassembled by any of ∼100 currently known deubiquitinating enzymes (DUBs) that are encoded in the human genome. These DUBs belong to five distinct classes: four established cysteine protease families, and a class of metalloproteases.^2^ The function of many DUBs remains unclear at present, and the study of their function depends on the availability of suitable assay reagents.

**Figure 1:**
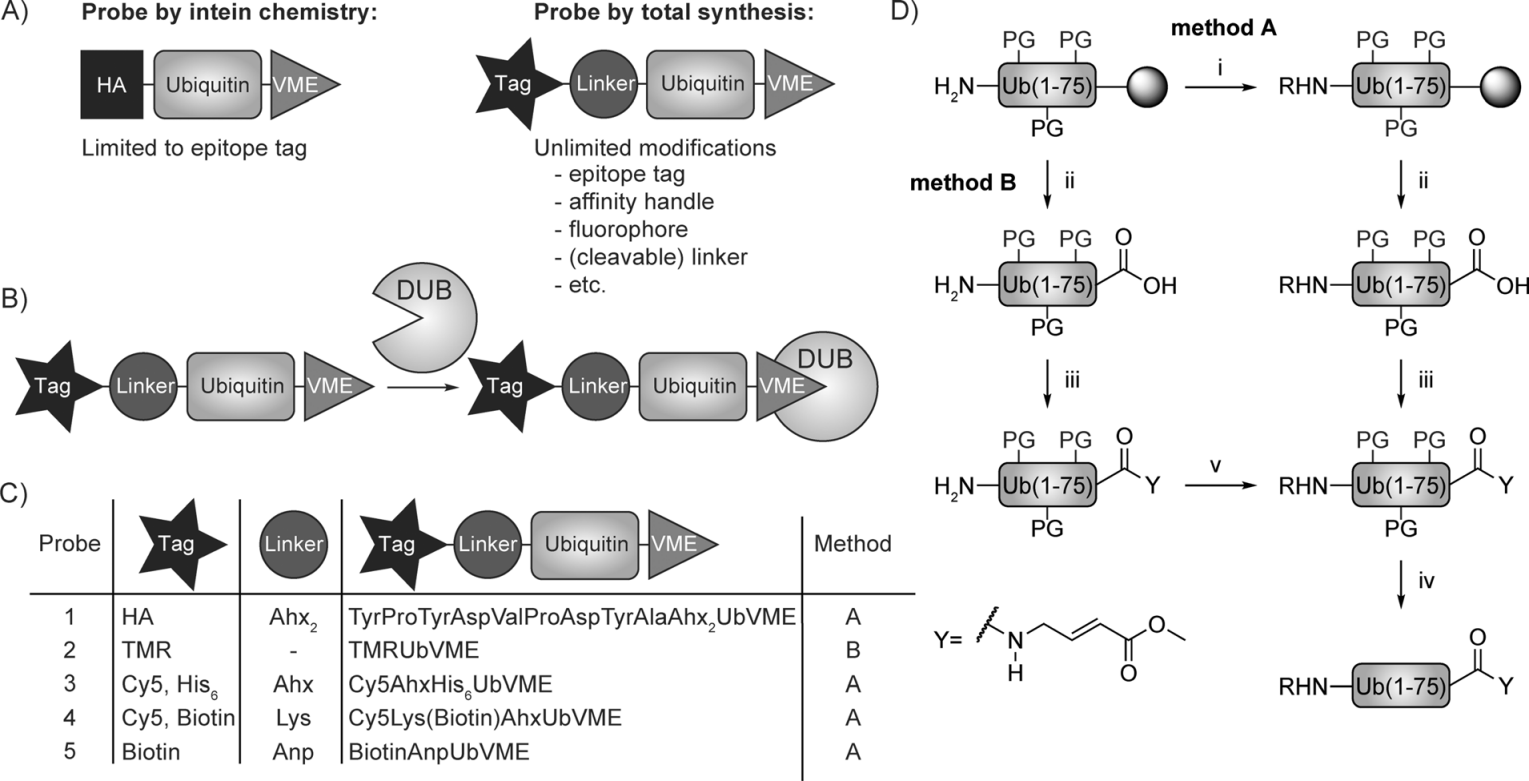
A) Classical DUB probes versus chemically synthesized probes. B) Ub-based probes react with the active-site cysteine residue present in most DUBs. C) Table of probes synthesized via the two reaction pathways. See Figure S2 for structures of incorporated tags and linkers. D) Synthesis scheme: two reaction pathways to chemically synthesize active-site-directed Ub-based probes i) 1. PyBOP, DIPEA, R-COOH, NMP, 16 h, RT; 2. Piperidine, NMP, 3×10 min., RT; ii) HFIP/DCM, 30 min, RT; iii) PyBOP, Et_3_N, GlyVME, DCM, 16 h, RT; iv) TFA, TIPS, H_2_O, 3 h, RT; v) PyBOP, DIPEA, TMR, DCM, 16 h, RT. PG=protecting group.

Current activity-based probes that target DUBs are based on the Ub sequence and are equipped with a reactive C-terminal warhead, for example, vinyl methyl ester (VME), and an N-terminal epitope tag (Figure 1 A), and they react with the active site cysteine residue that is present in most DUBs,^3^ as shown in Figure 1 B. These probes can be used to visualize the activity of multiple DUBs in a single experiment and they have proved indispensable in the search for novel DUBs, including a novel class of ovarian tumor (OTU)-domain-containing DUBs^3^ and DUBs encoded by pathogens such as the herpes virus,^4^ apicomplexan parasites *Toxoplasma gondii*^5^ and *Plasmodium falciparum*,^6^ and the nematode *Trichinella spiralis*.^7^ In addition, with the Ub system arising as a novel pool of potential drug targets,^8^ Ub-based DUB probes have proven instrumental in monitoring the potency and specificity of small-molecule DUB inhibitors in competition experiments.^9^ Current activity-based probes are limited to those with the HA epitope tag, and the classical preparation of DUB probes takes advantage of intein chemistry to generate intermediate thioesters that are converted into active-site-directed probes and more recently into probes with C-terminally extended isopeptide warheads.^10^ Intein chemistry tends to be laborious, the required thioesters are frequently partially hydrolyzed, and the amount of material that can be obtained is often limited. Secondly, intein-mediated expression and ligation methods are largely limited to those amino acids that can be encoded genetically, although recent advances have been made that allow the modification of the Ub sequence by using biological genetic code expansion methods.^11^ Synthetic methods can overcome these limitations. We recently reported the total linear synthesis of Ub,^12^ thereby allowing convenient control over additional residues that can be incorporated. This synthetic route can be scaled up to generate larger amounts of material than is feasible when using intein-based techniques.

Here, we describe how linear Ub synthesis can be used to devise new Ub-based activity probes by decorating the Ub N terminus with dyes, affinity handles (epitope tag, biotin), spacers, and cleavable linkers, while selectively adding a C-terminal active-site directed moiety (Figure 1 A). We demonstrate the value of these probes in activity-profiling experiments and the assessment of small-molecule DUB inhibitor specificities. Furthermore, we show that the installation of pull-down handles and selectively cleavable linkers facilitates isolation and DUB release procedures.

## Results and Discussion

### Linear chemical synthesis of active-site-directed ubiquitin-based probes

We based the synthetic route towards novel UbVME probes on the linear solid-supported total synthesis of Ub that we reported recently,^12^ which makes use of four pseudoproline building blocks and two dimethoxybenzyl (Dmb) dipeptides to prevent folding and/or aggregation of the growing peptide chain on-resin. In that study, we reported that a minimum of four of these building blocks was required for a synthesis that was sufficiently productive. A recent report claimed, without further experimental evidence, to have further optimized linear Ub synthesis by using only two of these building blocks.^13^ However, a careful comparison (Figure S1 in the Supporting Information) confirms that the use of six such building blocks provides Ub products of superior quality, as we reported previously.^12^

**Figure 2:**
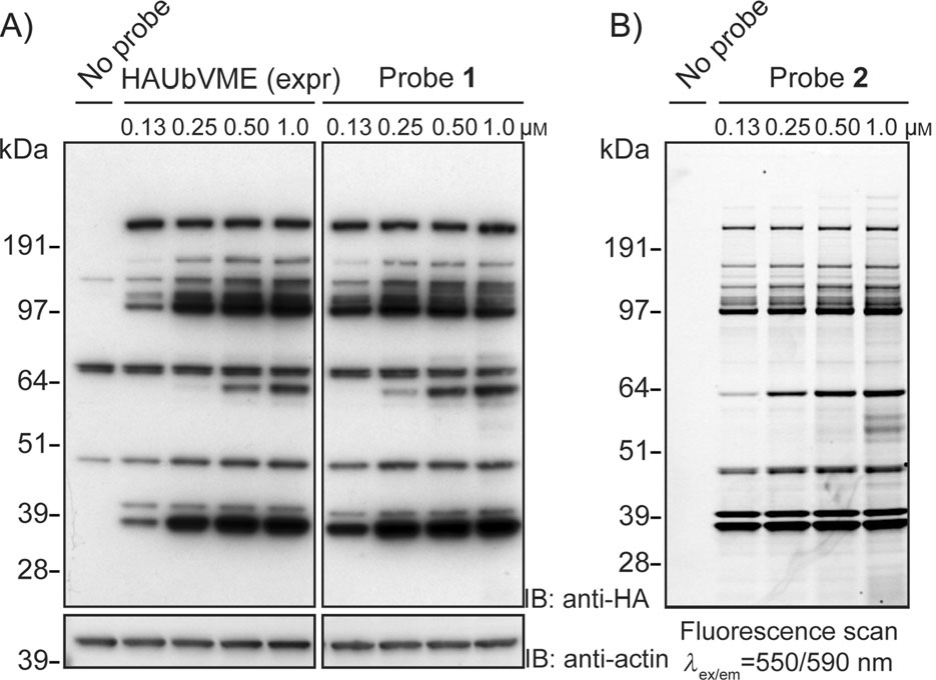
A) EL4 cell lysate incubated with classical probe HAUbVME (HAUbVME (expr)) obtained by the conventional intein method, and with the chemically synthesized, HA-tagged probe 1. Proteins were separated by SDS-PAGE and analyzed by immunoblotting. Actin, loading control. B) EL4 lysate incubated with fluorescent probe 2. Proteins were separated by SDS-PAGE and analyzed by in-gel fluorescence scanning.

We first focused on the total chemical synthesis of the classical epitope-tagged DUB probe HAUbVME, previously generated by intein chemistry,^3^ as a benchmark. Our synthetic approach towards HA-tagged UbVME **1** (Figure 1 C) is outlined in Figure 1 D (Method A). The Ub(1–75) sequence (C-terminal Gly residue omitted) was built up on a hyper-acid-labile trityl resin. Subsequently, two 6-amino hexanoic acid (Ahx) residues and the amino acids of the HA-tag (YPYDVPDYA) were sequentially introduced to the N terminus of the side-chain-protected, resin-bound peptide. The Ahx residues were incorporated to favor accessibility to anti-HA antibody. After mild acidic cleavage from the resin, glycine vinyl methyl ester (GlyVME) was coupled to the free C terminus of the partially protected peptide in solution. Subsequent deprotection and HPLC purification afforded HA-tagged UbVME **1** in 16 % overall yield following purification. To evaluate application of chemically synthesized HA-tagged UbVME **1**, EL4 cell lysate was incubated with either the classical HA-tagged probe (prepared by intein chemistry) or synthetic probe **1**, and DUB activity was visualized by immunoblotting (Figure 2 A). Identical labeling profiles were observed, thus showing that chemically synthesized probes can be successfully used to label DUBs.

We then turned our attention to the generation of a probe holding a fluorophore to allow direct in-gel fluorescence scanning. Fluorescence imaging is not only faster than classical immunoblotting techniques, it also circumvents unspecific background labeling due to cross-reactivity of the antibodies used for immunoblotting. Following condensation of GlyVME at the C terminus, the fluorophore 5-carboxytetramethylrhodamine (TMR) was introduced at the N terminus of the Ub-based probe (Method B, Figure 1 D). Removal of protective groups followed by purification yielded probe **2** (Figure 1 C) in 11 % overall yield following purification. The synthetic methodology shown in Figure 1 D conveniently allowed incorporation of a range of single or multiple handles, including other fluorophores such as Cy5 in probes **3** and **4**, pull-down handles such as a hexahistidine tag in probe **3**, biotin in probes **4** and **5**, and a photocleavable linker in probe **5** (Figure 1 C). Total yields of 10–20 % following purification were obtained for the synthesis of these UbVME probes after synthesis and HPLC purification. Liquid chromatography profiles and mass spectrometric characterizations of probes **1–5** are shown in Figures S3–S9.

### Fluorescent UbVME probe allows fast detection of DUBs in cell lysate

To investigate whether probe **2** can be used to profile DUB activity with similar sensitivity and detection limits as the classical probe HAUbVME, EL4 cell lysate was assayed with increasing probe concentrations (Figure 2 A and B). Labeled DUBs were visualized either by in-gel fluorescence (Figure 2 B), or by chemilumiscence following immunoblotting (Figure 2 A). Similar DUB labeling patterns were observed for probe **2** and HA-tagged UbVME, and labeling with the probes was dose-dependent (Figure 2 A and B). Labeling of DUBs in cell lysate with probe **2** allowed direct in-gel fluorescence scanning, thereby resulting in a much faster analysis than with immunoblotting techniques. Furthermore, probe **2** appeared slightly more sensitive, and revealed a few less-abundant or less-active DUBs that were not easily revealed by HA-tagged UbVME. In addition, with probe **2** a clearer picture was obtained, and bands could be distinguished with higher resolution, likely because of the direct nature of the readout. When near-infrared fluorescent secondary antibodies were used, higher resolution pictures were obtained (Figure S10 A), compared to visualization by chemiluminescence (Figure 2 A). Still, superior pictures for labeled cell lysate were obtained by using fluorescent probe **2** (Figures 2 B and S10 B).

### Biotin-tagged dual-activity probes for labeling and affinity pull-down of proteins

**Figure 3:**
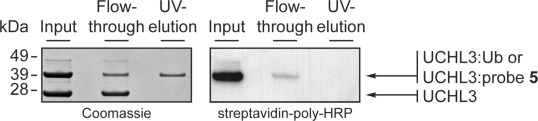
Affinity pull-down of UCHL3 by using photocleavable probe 5. Proteins were separated by SDS-PAGE and analyzed by Coomassie staining and by western blotting followed by staining with streptavidin–poly-HRP. Release of labeled UCHL3 by UV is shown (left panel), whereas photocleavage was confirmed by the absence of a biotin tag (right panel).

Having shown that N-terminal tags can be introduced onto UbVME probes without significantly changing DUB labeling patterns, two tandem-tagged activity probes were designed by combining a biotin-tag (to allow affinity pull-down of labeled DUBs) with either a fluorophore (for fluorescence imaging, probe **4**) or a photocleavable moiety (for catch-and-photorelease purposes; probe **5**). To investigate whether the biotin tag of probe **4**, when bound to a DUB, was accessible for capture by streptavidin, DUBs present in EL4 lysate were labeled with probe **4** and visualized by either in-gel fluorescence scanning or by western blotting (Figure S11). Congruent DUB labeling patterns were observed with both visualization methods, thus showing that a biotin-tagged DUB-activity probe can be used to pull down DUBs from cell extracts. In a next step, the catch-and-photorelease principle was evaluated by the immobilization of recombinant UCHL3 (a 26 kDa protein) labeled with probe **5**. Loaded neutravidin resin was exposed to UV light, and input, flow through, and UV-elution samples were analyzed by SDS-PAGE (Figure 3). UCHL3 labeled with probe **5** yielded an additional band that migrated at approximately 38 kDa. Coomassie staining and blotting with streptavidin–poly-HRP verified biotinylation of UCHL3 by labeling with probe **5** (“input” lanes, Figure 3). Coomassie staining showed that the UV-elution sample contained UCHL3:probe conjugate (Figure 3, left), while the absence of a biotin tag (Figure 3, right) verified the catch-and-photorelease principle.

### Assessment of DUB inhibitor potency by using a fluorescent UbVME probe

**Figure 4:**
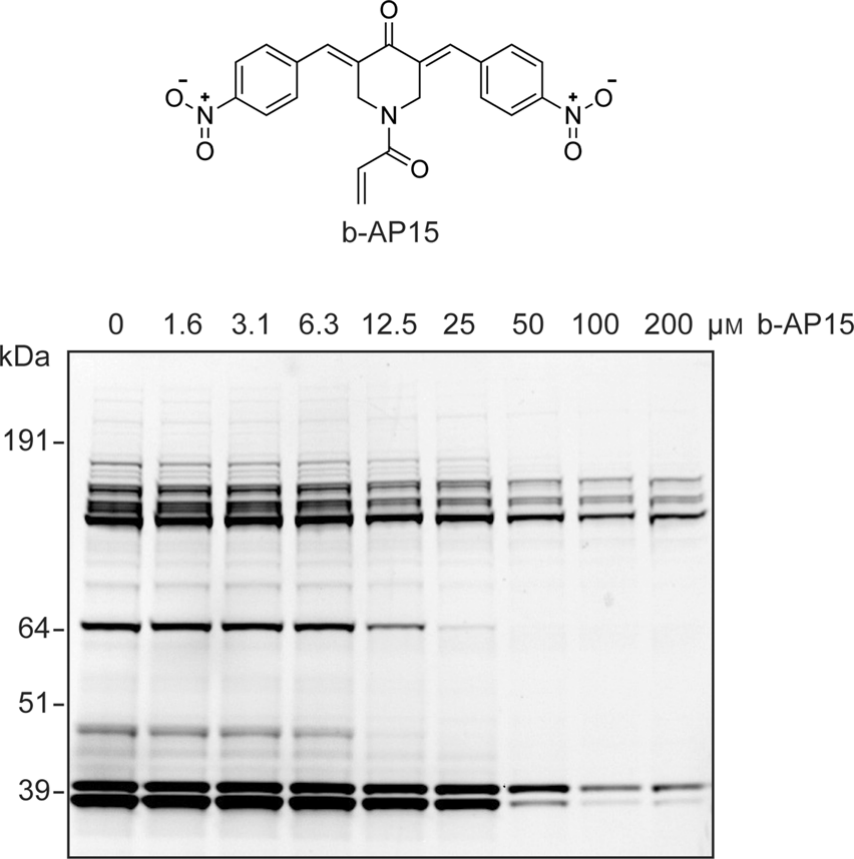
EL4 lysate was incubated with the indicated concentrations of small-molecule DUB inhibitor b-AP15 (top). Subsequently, lysate was labeled with probe 2. Proteins were separated by SDS-PAGE and the residual DUB activity was visualized by in-gel fluorescence scanning (bottom).

Ub-based probe competition experiments have recently been reported in the assessment of DUB inhibitor potency by using HA-tagged probe.^9^ As fluorescent probe **2** allows rapid in-gel detection of DUBs, DUB inhibitor profiles can be obtained now in a more high-thoughput manner. To test whether fluorescent probe **2** can be used for testing the potency and selectivity of DUB inhibitors, four compounds were selected that inhibit DUBs associated with cell growth and tumor progression: b-AP15,^14^ Compound A,^15^ PR-619,^9a^ and WP1130.9b, 16 The potency of each selected DUB inhibitor was examined in a competitive activity-based assay in which EL4 cell extract was incubated with a range of inhibitor concentrations and subsequently labeled with probe **2** (Figures 4 and S12). The resulting gels display a dose-dependent reduction of probe labeling for all four inhibitors and all appeared to block a wide panel of DUBs when using increasing inhibitor concentrations.

### Assaying the effect of genetic and cell biological manipulations on cellular DUB activity

Because fluorescent UbVME probes offer a direct means to assay DUB activity in cell lysate, they may be utilized to monitor the functional outcomes of genetic and cell biological manipulations of specific DUBs. USP14 is an abundant DUB that reversibly associates with the proteasome and trims Ub chains before substrate degradation. Inhibition of USP14 by the small molecule IU1 has been shown to enhance proteasome activity, which has been suggested as a novel therapeutic strategy.^17^ USP14 (∼56 kDa) migrates as a 65 kDa fluorescent band once attached to the probe. MelJuSo cells were treated with a pool of four siRNA oligos that target USP14 (Figure S13, lane 2), individual oligos (Figure S13, lanes 3 and 4) or control siRNA (Figure S13, lane 1). A decrease in USP14 reactivity with probe **2** reflects loss of functional USP14 from respective lysates. No appreciable loss of other probe-reactive bands was detected in the same samples in response to anti-USP14 siRNA.

**Figure 5:**
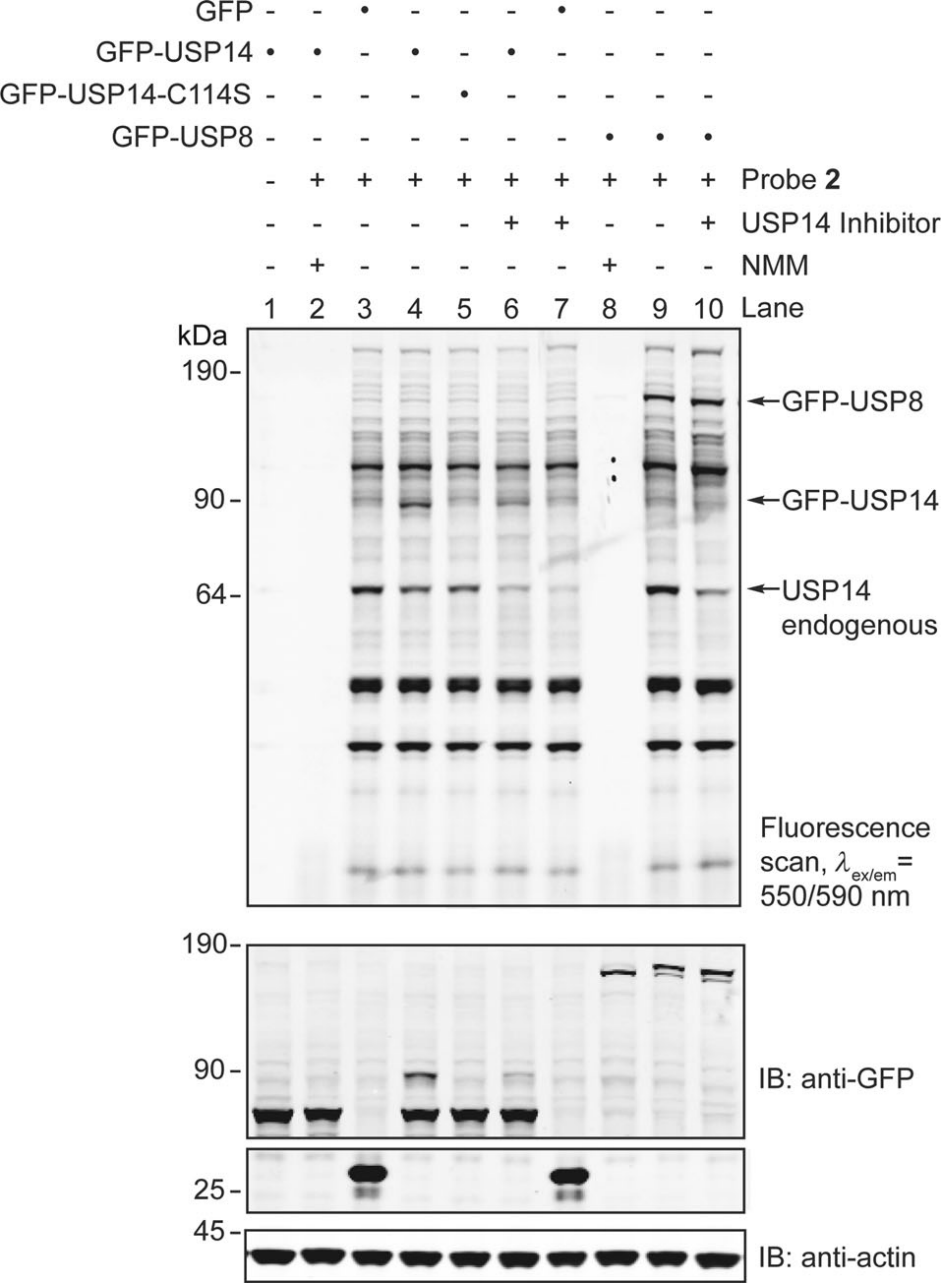
GFP, GFP-USP14, GFP-USP14 catalytic mutant, and GFP-USP8 were overexpressed in MelJuSo cells, and lysates were incubated in the absence or presence of inhibitor IU1 or NMM, before labeling with probe 2. Proteins were separated by SDS-PAGE before fluorescence scanning or western blotting followed by profiling with the indicated antibodies. Actin, loading control. IB=immunoblotting.

As with DUB depletion, probe **2** can be used to profile the activity of DUBs expressed ectopically in their normal cellular environment, where endogenous post-translational modifications and spatiotemporal localization are maintained. Furthermore, in combination with standard immunoblotting techniques, probe **2** enabled quantitative assessment of the proportion of reacted enzyme. For instance, in the presence of probe **2**, over-expression of GFP-tagged USP14 yielded an additional fluorescent band at 90 kDa not observed when expressing GFP alone, or catalytic point-mutant USP14-C114S (Figure 5, lanes 3–5, top). Accordingly, immunoblotting for GFP revealed a shift of approximately 10 kDa for GFP-USP14 in the presence of probe **2**, but not for the C114S mutant (Figure 5, lanes 1, 4, and 5, anti-GFP IB). Specific inhibition of both endogenous and GFP-tagged USP14 was observed in cells treated with compound IU1 (Figure 5, lanes 4, 6 and 7),^17a^ whereas reactivity of other abundant endogenous DUBs or over-expressed GFP-USP8 remained insensitive to IU1 (Figure 5, lanes 9 and 10). Conversely, all probe-reactive bands, including GFP-USP8, were completely inhibited in the presence of the cysteine alkylating agent *N*-methylmaleimide (NMM, Figure 5, lanes 2 and 8).

### Visualization of differential DUB activity profiles of cancer cells

Probe **2** also allows a fast and sensitive characterization of differential DUB activity patterns, for instance observed in various cancer cell lines. DUB profiling can be used to identify tumor-specific DUBs and their inhibitors, thus contributing to the definition of new targets in cancer therapy. In healthy individuals expression of UCHL1 is limited to neurons and testes,^18^ while overexpression of UCHL1 has been shown in a variety of cancers.^19^ For example, UCHL1 is markedly expressed in lung cancer cell lines and primary lung tumors, but not in normal lung tissue.^20^ UCHL1 has recently also been shown to promote cancer metastasis in prostate cancer cells.^19^ The function of UCHL1 is not fully understood, although UCHL1 has been shown to enhance invasive potential in vitro and in vivo.^21^

**Figure 6:**
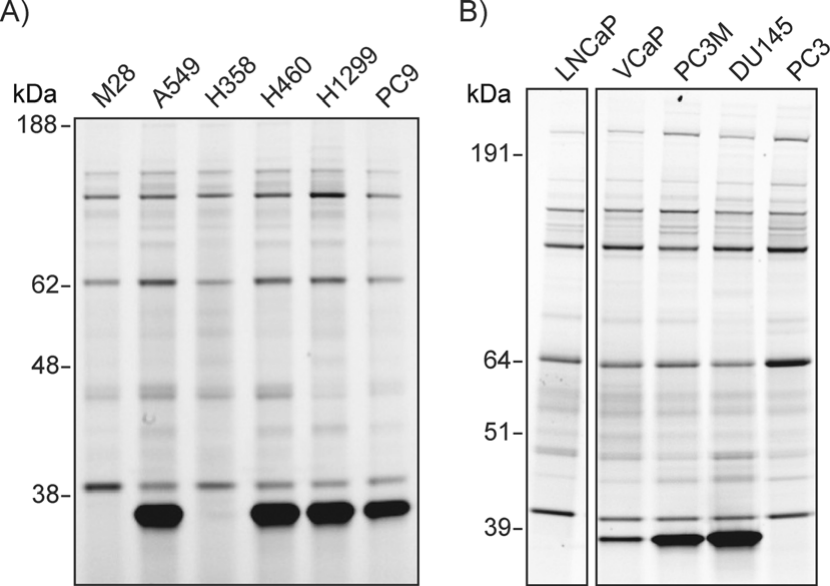
Visualization of DUB activity in cancer cell lines. A) In a panel of lung cancer cell lines, DUB activity profiles were examined by treating lysates with probe 2. UCHL1 is highly expressed in cell lines A549, H460, H1299, and PC9. B) In a panel of prostate cancer cell lines probed with 2, UCHL1 is highly expressed in cell lines VCaP, PC3M, and DU145.

We used probe **2** to visualize the activity of UCHL1 in a panel of lung and prostate cancer cell lines. In the various lung cancer cell lines tested, we observed high UCHL1 activity in cell lines H1299,^22^ H460,^23^ PC9,^24^ and A549,^25^ but no UCHL1 activity was observed in cell lines M28 or H358^21a^, 22 (Figure 6 A), which is consistent with reported literature. In the prostate carcinoma cell lines we observed high activity of UCHL1 in VCaP, DU145,^19^ and PC3M cells, but no activity in PC3^24^ or LnCap cells^26^ (Figures 6 B and S14). UCHL1 expression was confirmed by immunoblotting (Figure S15). Recently, UCHL1 was demonstrated to contribute to the highly metastatic character of DU145 cells (derived from brain metastatic sites).^19^ Interestingly, we observed high UCHL1 activity in PC3M cells, a more metastatic variant of the PC3 cell line that does not display UCHL1 activity, further supporting a link to metastasis.

## Conclusions

We have shown that Ub-based probes can be synthesized chemically with great ease and in large amounts, and that this method can be adapted to include modules that fulfill specific experimental requirements. A great variety of building blocks can now be easily incorporated into the Ub sequence, such as natural and unnatural amino acids, fluorescent tags, affinity handles, spacers, and cleavable linkers. We have demonstrated that similar labeling patterns are observed when labeling cell extract with either epitope-tagged probe obtained by intein chemistry or chemically synthesized epitope-tagged probe. We could easily incorporate additional affinity handles and selective cleavage moieties thereby enabling affinity pull-down and subsequent mild UV-activated release of DUBs. Incorporation of a fluorescent tag allowed fast direct in-gel fluorescence scanning, and we have shown that fluorescent UbVME is a sensitive and convenient tool for the rapid profiling of DUB inhibitor potency and specificity. For example, we were able to demonstrate that amongst the five reported DUB inhibitors tested here, IU1 was selective for USP14, while the other inhibitors can be regarded as broad-spectrum DUB inhibitors. Genetic and cell biological manipulation of cellular DUB activity was successfully tracked by using a fluorescent probe, as illustrated by the visualization of depletion of USP14 and the overexpression of GFP-tagged USP14 and USP8. Direct comparison of differential DUB activity profiles amongst a panel of cancer cell lines might find application as a rapid diagnostic tool to predict the outcome of DUB inhibitor therapy. In conclusion, the DUB-activity probes reported here constitute an important addition to the laboratory toolkit in ubiquitin signal transduction research.

## Experimental Section

**General:** All chemicals used in this study were purchased from Biosolve (Valkenswaard, The Netherlands) or Sigma–Aldrich, unless otherwise indicated, and at the highest commercially available grade. Peptide building blocks were purchased from Novabiochem (EMD Millipore) and glycine-functionalized trityl resin (TentaGel R TRT-Gly Fmoc) from Rapp Polymere (Tübingen, Germany). All chemicals and solvents were used as received. Cy5,^27^ TMR,^28^ (*E*)-methyl-4-aminobut-2-enoate (GlyVME),^29^ inhibitor compound A, b-AP15^30^ and PR-619^9a^ were synthesized according to previously established methods. Compound WP1130 was purchased from Selleck Chemicals (Houston, TX). Ubiquitin carboxyl-terminal esterase L3 (UCH-L3) was produced recombinantly according to published procedures.^31^ Preparative HPLC was performed on a Prominence HPLC system (Shimadzu) equipped with a Atlantis T3 column (10×150 mm, 5 μm; Waters) by using two mobile phases: A (TFA (0.1 %) in water) and B (formic acid (0.1 %) in acetonitrile): flow rate, 7.5 mL min^−1^; run time, 35 min; column temp., 40 °C; gradient: 0–5 min, 5 % B; 5–8 min, →25 % B; 8–30 min, →60 % B; 30–33 min, →95 % B; 33–35 min, 95 % B. Analytical HPLC was performed on a 1525EF Binary HPLC pump (Waters) equipped with a 2487 Dual λ Absorbance Detector. Samples were run over an Atlantis DC18 column (6.4×150 mm, 10 μm; Waters) with two mobile phases: A (TFA (0.05 %) in water) and B (TFA (0.05 %) in acetonitrile); gradients: 0–1 min, 1 % B; 1–13 min, →90 % B; 13–16 min, 90 % B; 16–17 min, →1 % B; 17–25 min, 1 % B; or 0–5 min, 5 % B; 5–30 min, →95 % B; 30–35 min, 95 % B; 35–40 min, →5 % B; 40–45 min, 5 % B. LC-MS measurements were performed on a system equipped with a Alliance 2795 Separation Module (Waters), 2996 Photodiode Array Detector (190–750 nm), and LCT Orthogonal Acceleration Time of Flight Mass Spectrometer. Samples were run over a kinetix C18 column (2.1×50 mm, 2.6 μm; Phenomenex, Torrence, CA), with flow rate 0.8 mL min^−1^, runtime 6 min, column temp. 40 °C, and two mobile phases: A (acetonitrile(1 %) and formic acid (0.1 %) in water) and B (water (1 %) and formic acid (0.1 %) in acetonitrile); gradient: 0–0.5 min, 5 % B; 0.5–4 min, →95 % B; 4–5.5 min, 95 % B. Data processing was performed with MassLynx Mass Spectrometry Software 4.1 (deconvolution with Maxent1 function; Waters). SDS-PAGE was performed on 4–12 % NuPAGE Novex Bis-Tris Mini-gels (Invitrogen) and run in MOPS buffer at 170 V, unless stated otherwise. In-gel fluorescence scans were obtained by using a ProXPRESS 2D Proteomic imaging system (Perkin–Elmer) with a resolution of 100 μm and exposure time of 60 s, with filter settings (*λ*_ex_/*λ*_em_) 550/590 nm (TMR) or 625/680 nm (Cy5). Chemiluminescence (ECL western blotting detection kit; GE Healthcare) was visualized on a Chemidoc XRS+ System with Image Lab software (Bio-Rad, Hercules, CA). Immunoblots stained with fluorescent antibodies were visualized by using an Odyssey Infrared Imaging System (LI-COR, Lincoln, NE).

**Cell culture:** EL4 cells, lung cancer cells M28, A549, PC-9, H358, H460, and H1299, and prostate cancer cells PC3, PC3M, DU145, VCaP, and LNCaP were grown in Gibco RPMI 1640 medium (Life Technologies) supplemented with fetal calf serum (FCS, 10 % *v*/*v*) at 37 °C in a 5 % CO_2_ atmosphere. EL4 growth medium was supplemented with penicillin (100 U mL^−1^) and streptomycin (100 μg mL^−1^). The growth medium for the prostate cancer cell lines was supplemented with l-glutamine (2 mm). MelJuSo cells were grown in Gibco IMDM medium (Life Technologies) supplemented with FCS (10 %) at 37 °C in a 5 % CO_2_ atmosphere. Prostate cancer cell lines PC3, PC3M, DU145, and LNCaP were obtained from the American Type Culture Collection (Manassas, VA). VCaP cells were kindly obtained from Dr. Guido Jenster (ECMC Rotterdam).

**Linear chemical synthesis of active-site directed Ub-based probes**

*Method A (for probes **1**, **3**, **4**, and **5**):* The Ub(1–75) peptide sequence with a free N terminus but with side chains protected, was synthesized (25 μmol scale) on a trityl resin by following Fmoc solid-phase peptide synthesis procedures as described,^12^ with minor modifications. Briefly, for the first 30 cycles, couplings were performed in *N*-methylpyrrolidone (NMP) for 40 min by using PyBOP (4 equiv), *N*,*N*-diisopropylethylamine (DIPEA) (8 equiv), and protected amino acid (4 equiv). Fmoc protecting groups were removed by treating the resin with piperidine (20 %) in NMP (2×3 min and 1×5 min). For Leu43, Lys48, Leu50, and Glu51, double coupling was applied (2×40 min). For cycles 31–40, the coupling time was extended to 60 min and double couplings were applied for Asp32, Lys33, Pro 37, and Pro38. Fmoc deprotection was performed by using piperidine (20 %) in NMP (4×3 min). After cycle 40, double couplings were performed for all amino acids. Pseudoproline building blocks Fmoc-L-Leu–L-Thr(ΨMe,Mepro)-OH (replacing Leu8–Thr9), Fmoc-L-Ile–L-Thr(ΨMe,Mepro)-OH (replacing Ile13–Thr14), Fmoc-L-Leu–L-Ser(ΨMe,Mepro)-OH (replacing Leu56–Ser57), Fmoc-L-Ser(*t*Bu)–L-Thr(ΨMe,Mepro)-OH (replacing Ser65–Thr66), and Dmb dipeptides Fmoc-L-Ala–(Dmb)Gly-OH (replacing Ala46–Gly47) and Fmoc-L-Asp(O*t*Bu)–(Dmb)Gly-OH (replacing Asp52–Gly53) were coupled by using single couplings for 90 min. The N-acetyl capping steps that we applied in previous syntheses^12^ were omitted here. Double couplings were applied for the Fmoc-protected amino acids of the HA-tag and His_6_-tag. Carboxy-functionalized Cy5, biotin, or Fmoc-protected Ahx linker (Fmoc-Ahx-OH), Fmoc-protected 3-amino-3-(2-nitrophenyl) propionic acid (Fmoc-Anp-OH) or Fmoc-protected lysine-containing biotin coupled to the ε amine (Fmoc-Lys(Biotin)-OH; Figure S2) were coupled to the N terminus of Ub on resin by using building block (4 equiv), benzotriazol-1-yl-oxytripyrrolidinophosphium hexafluorophosphate (PyBOP; 4 equiv), and DIPEA (8 equiv) in NMP at ambient temperature for 16 h. Fmoc groups were removed as described.^12^ N-terminally functionalized Ub conjugates were removed from the resin by using 1,1,1,3,3,3-hexafluoropropan-2-ol (HFIP) as described.^12^ Gly-VME (10 equiv) was coupled to the C terminus of Ub by using PyBOP (5 equiv), triethylamine (Et_3_N) (20 equiv) in DCM (5 mL) and stirred for 16 h at ambient temperature. Excess Gly-VME was removed by washing the DCM solution with 1 M KHSO_4_. The organic layer was dried with Na_2_SO_4_ and concentrated to dryness in vacuo. To remove the side-chain protecting groups, the residue was taken up in trifluoroacetic acid/triisopropylsilane/water (5 mL; 95:2.5:2.5) and stirred for 3 h at ambient temperature. The reaction mixture was added to a falcon tube containing ice-cold pentane/diethyl ether (1:3; 40 mL), upon which the product precipitated. The precipitate was isolated by centrifugation (1500 *g*, 6 min, 4 °C) and washed by three cycles of resuspension in ice-cold diethyl ether and centrifugation. Finally, the pellet was taken up in water/acetonitrile/acetic acid (65:25:10), frozen, and lyophilized.

*Method B (for probe **2**):* As TMR contains two carboxylic acid groups, subsequent coupling of GlyVME to the peptide would lead to the attachment of two GlyVME moieties, one at the C terminus and one at the TMR carboxylate. To prevent this, Ub(1–75) with a free N terminus but protected side chains was cleaved from the resin by using HFIP as described,^12^ and GlyVME was coupled to the C terminus in solution as described above, before condensation of TMR (4 equiv) to the N terminus, by using PyBOP (4 equiv) and DIPEA (10 equiv) in DCM (5 mL), and stirring for 16 h at ambient temperature. The reaction mixture was concentrated to dryness in vacuo. Removal of side chain protecting groups was performed as for Method A.

All resulting probes were subsequently purified by preparative HPLC. For further applications, probes were dissolved (to 25 μm) in sodium acetate buffer (50 mm, pH 4.5) containing DMSO (5 %). Liquid chromatography profiles and mass spectra of all probes synthesized are shown in Figures S3–S9.

**Preparation of cell extracts and labeling with Ub-based probes:** Cells were lysed by sonication in lysis buffer (Tris (50 mm), sucrose (250 mM), MgCl_2_ (5 mM), DTT (1 mM)) supplemented with CHAPS (0.5 %) and NP40 (0.1 %), and clarified by spinning (16 000 *g*, 10 min, 4 °C). For lysis of prostate cancer cell lines 1× Complete Protease Inhibitor Cocktail (Roche) was added to the lysis buffer. Typically, labeling experiments were performed in lysis buffer (25 μL) containing protein extract (1 mg mL^−1^) and Ub-based probe (1 μm), unless otherwise indicated. The pH was neutralized by adding NaOH (50 mm, 2 equiv (*v*/*v*) relative to probe). Labeling reactions were incubated for 30 min at ambient temperature before being terminated by addition of reducing sample buffer and heating (70 °C, 10 min). Activity-based protein profiling of DUB inhibitors was performed in the presence of DMSO (5 %). Extracts were preincubated with compound at the indicated concentrations and times, before the addition of probe and a further incubation for 15 min at ambient temperature. Proteins were resolved by SDS-PAGE. Following in-gel fluorescence scanning, gels were transferred onto PVDF membranes (1 h, 15 V) and blotted by using mouse anti-HA (12A5; Roche), mouse anti-β-actin (Sigma–Aldrich), or streptavidin–poly-HRP (Sanquin, Amsterdam, the Netherlands). Where necessary, HRP-conjugated rabbit anti-mouse was used as a secondary antibody (P0161; Dako, Glostrup, Denmark), and immunoblots were visualized by chemiluminescence. Alternatively, gels were transferred onto nitrocellulose membranes (1 h, 15 V) and blotted with rabbit anti-HA (Sigma–Aldrich) and mouse anti-β-actin (Sigma–Aldrich) in combination with fluorescent secondary antibodies goat anti-mouse IRDye 680LT and goat anti-rabbit-IRDye 800CW (LI-COR), and visualized by fluorescence scanning.

**Affinity pull-down and photoactivated release of UCH-L3:** In buffer A (40 μL, phosphate buffer (100 mm, pH 7.1), NaCl (150 mM)) containing DMSO (2.5 %), UCH-L3 (25 μg) was incubated with probe **5** (25 μm) for 1 h at 37 °C. The reaction mixture was added to buffer A (30 μL) and High Capacity Neutravidin resin (20 μL, Thermo Scientific) that was washed (5×) with buffer A (100 μL), and incubated for 3.5 h at 37 °C. The beads were washed extensively with buffer A (5× 70 μL) and buffer B (Tris**⋅**HCl (50 mm, pH 7.0), NaCl (300 mm), SDS (1 %); 4×70 μL). Beads were taken up in buffer A (70 μL) containing DTT (1 mm), and transferred to a 96-well plate (Black Flat Bottom Polystyrene, nonbinding surface; Corning, Corning, NY). The plate was shaken at 4 °C for 1 h while exposed to UV (365 nm, 15 W; Uvitec, Cambridge, UK). Input, flow through, and elution samples were resolved by reducing SDS-PAGE (12 %, MES). One gel was stained with Coomassie, while a duplicate gel, run simultaneously, was used for blotting with streptavidin–poly-HRP as described above.

**Genetic and cell biological manipulations affecting cellular DUB activity:** Knockdown of USP14 in MelJuSo cells was achieved by using either a pool of four siGENOME oligonucleotides or individual oligos siUSP14-1 (GCAUA UCGCU UACGU UCUA) or siUSP14-2 (GGAGU UACCA UGUGG AUUG; Dharmacon/Thermo Scientific) at a final concentration of 50 nm, delivered by transient transfection with DharmaFECT 4 (Dharmacon) according to manufacturer's instructions. Cells were grown in Falcon six-well tissue culture plates (BD Biosciences). Samples were harvested 72 h following transfection by scraping on ice in lysis buffer (100 μL). Lysates were briefly sonicated and clarified (10 000 rpm, 10 min, 4 °C). Lysate preparation (1.87 mg mL^−1^) in lysis buffer (30 μL) was incubated with probe **2** (1.67 μm) for 15 min at ambient temperature. Reactions were terminated, and samples were resolved by SDS-PAGE and analyzed as described above.

For overexpression of GFP-tagged DUBs in cells, wild type USP14 was subcloned from pDEST-USP14 (Addgene, Cambridge, MA) into eGFP-C1 vector (Clontech, Mountain View, CA) at XhoI/EcoRI restriction sites by using standard protocols. Mutagenesis (C114S) to generate a catalytically inactive USP14 was performed according to standard protocols by using TurboPfu DNA polymerase (Stratagene/Agilent Technologies) with the forward (CTT GGT AAC ACT TCT TAC ATG AAT GCC) and reverse (GGC ATT CAT GTA AGA AGT GTT ACC AAG) primers (Invitrogen/Life Technologies). The GFP-USP8 construct was generously provided by Dr. Sylvie Urbé (University of Liverpool, UK). DNA delivery into MelJuSo cells was performed in 60 mm tissue culture plates by using Lipofectamine2000 (Invitrogen) according to manufacturer's instructions. Samples were harvested 24 h following transfection in buffer (0.25 mL), and lysates were prepared as indicated above. DMSO alone or with dissolved inhibitors was added to lysis buffer (10 μL) supplemented with CHAPS (0.5 %) and NP40 (0.1 %) (DMSO <6 % of total reaction volume) and mixed with lysate preparation (20 μL). Final inhibitor concentrations were: *N*-methyl maleimide (NMM) 4 mM, IU1 100 μM. Samples were incubated for 30 min at ambient temperature, and probe **2 (**2 μL, 25 μm) was then added for 5 min at ambient temperature. Samples were analyzed as described above. Following fluorescence scanning, gels were transferred onto nitrocellulose membranes (2 h, 300 mA) and immunoblotted with rabbit anti-GFP serum^32^ and mouse anti-β-actin (Sigma–Aldrich) in combination with fluorescent secondary antibodies (goat anti-mouse IRDye 680LT and goat anti-rabbit IRDye 800CW, LI-COR). Immunoblots were visualized by fluorescence scanning.
